# Molecular and Translational Research on Colorectal Cancer

**DOI:** 10.3390/ijms21114105

**Published:** 2020-06-09

**Authors:** Alessandro Passardi, Emanuela Scarpi, Paola Ulivi

**Affiliations:** 1 Medical Oncology Unit, Istituto Scientifico Romagnolo per lo Studio e la Cura dei Tumori (IRST) IRCCS, Via P. Maroncelli 40, 47014 Meldola (FC), Italy; alessandro.passardi@irst.emr.it; 2 Unit of Biostatistics and Clinical Trials, Istituto Scientifico Romagnolo per lo Studio e la Cura dei Tumori (IRST) IRCCS, Via P. Maroncelli 40, 47014 Meldola (FC), Italy; emanuela.scarpi@irst.emr.it; 3 Biosciences Laboratory, Istituto Scientifico Romagnolo per lo Studio e la Cura dei Tumori (IRST) IRCCS, Via P. Maroncelli 40, 47014 Meldola (FC), Italy

Colorectal cancer (CRC) is the third most frequently diagnosed cancer in the world. The completion of the human genome project and the availability of high-throughput technologies have led to a dramatic change in cancer research. Consequently, research into CRC has evolved to include translational and molecular oncology. Several studies have contributed to this, identifying the molecular mechanisms at the basis of CRC progression, defining pathways that influence treatment efficacy and resistance, and developing new tools and therapeutics to prevent or manage the disease more effectively. This open-access Special Issue has put together both original research and review articles on molecular and translational research into CRC.

CRC is a heterogeneous disease, with different subtypes identified and characterized by specific molecular and morphological alterations, such as RAS and BRAF mutations and kinase gene fusions. BRAF mutations, found in about 10% of CRC patients, define a particular subtype characterized by poor prognosis and resistance to chemotherapy. Recently, BRAF inhibitors have shown meaningful clinical activity in CRC, although inferior to that described for melanoma [1]. Kinase gene fusions account for less than 1% of all CRCs and identify another tumor subtype responding poorly to standard treatments, including EGFR inhibitors. Like BRAF mutations, they could potentially help identify a subgroup of patients who are most likely to benefit from selective targeted agents. However, the efficacy of these agents requires validation in prospective ad hoc clinical trials [2].

One of the most promising areas of translational research is the identification and validation of predictive and prognostic factors. Non-standard microsatellite instability appears to be a marker of poor prognosis in advanced CRC patients, with the worst outcomes observed in patients undergoing bevacizumab-based treatments [3]. Liquid biopsy has also been acknowledged as an important diagnostic, prognostic, and predictive biomarker. In addition, several circulating microRNAs have proven effective as predictive markers in CRC but few have been validated, indicating the need for further clinical studies on large patient cohorts to confirm their role in this setting [4]. 

Exosomes are an especially promising area of research as they are an excellent source of biomolecules that could serve as biomarkers or even therapeutic targets. Furthermore, exosomal noncoding RNAs could help in the detection of early-stage CRC as they have recently been shown to have higher sensitivity and specificity than CEA or CA19-9. Their prognostic potential has also been hypothesized [5]. Although the feasibility of exosome targeting therapy is still open to debate, innovative strategies to investigate this area include systemic exosome depletion, exosome-mediated circulating tumor cell capture, and exosome drug delivery [6]. Long noncoding RNAs (lncRNAs) play an important role in CRC growth and metastasis in that they function as competitive endogenous RNAs (ceRNAs), occupying the shared binding sequences of miRNAs, keeping the miRNAs apart, and modifying downstream target gene expression [7]. Within this context, the expression of four miRNAs has been found to be altered in CRC stem cells with respect to normal stem cells. In particular, the overexpression of miRNA92a appears to contribute to cancer stem cell origin [8].

The tumor microenvironment (TME), which includes macrophages, neutrophils, and fibroblasts, plays an important role in the initiation, progression, and invasion of CRC. Prostaglandin E2 (PGE2), a potent inflammatory mediator, has attracted the attention of researchers as it regulates immune cells and may promote the development of CRC. PGE2 has been found in various types of human malignancies including CRC, and is associated with poor prognosis. Thus, therapies targeting PGE2 or the specific downstream molecules of PGE2 signaling could be a promising approach [9]. Mucin expression and their polysaccharide components may also be involved in CRC development as differences in their expression between CRC tissue and its normal counterpart have been demonstrated [10]. 

Transforming growth factor-beta (TGF-β) signaling is well-known as an important pathway influencing tumorigenesis by modulating cell growth, differentiation, apoptosis, and homeostasis. Its role in both tumor cells and the TME is currently under investigation. The disruption of TGF-β signaling in CRC cells stimulates tumor formation, while its activation appears to promote invasion and metastasis. Its activation in the TME generally suppresses tumor immunity and supports cancer cell survival. This bidirectional phenomenon makes it difficult to develop drugs for the treatment of CRC [11]. TGF-β signaling also appears to be involved in the trafficking of extracellular vesicle protein content [12]. The metallothionein gene family is thought to play a role in CRC prognosis, and a four-gene signature composed of MT1F, MT1G, MT1L and MT1X has been shown to predict CRC patient outcome [13].

Epidemiological studies are underway to investigate several epigenetic biomarkers for their potential to predict outcome and response to treatment in CRC patients receiving neoadjuvant or adjuvant therapy. However, to date none has proven sufficiently robust to be introduced into a clinical setting [14]

In the field of immunotherapy, cancer vaccines are in the process of being developed for CRC. These vaccines work by reinforcing the immune system against tumor cells and have the advantage of having a favorable toxicity profile. However, results are still limited due to the slow effects of the vaccines and to the presence of immunosuppression [15].

Preclinical studies have identified potential targets for therapy, opening up new avenues for the development of novel treatment strategies [16,17,18,19,20]. Moreover, the regulation of EGFR expression by VEGFR signaling has been demonstrated [21], suggesting possible implications for the clinical use of anti-EGFR and anti-VEGF drugs. 

In conclusion, the rapidly growing field of molecular oncology is significantly impacting translational cancer research. The contributions of this Special Issue will thus serve to stimulate research into novel molecular targets in CRC to identify new strategies to improve diagnostic and therapeutic approaches.
